# Dexamethasone exerts profound immunologic interference on treatment efficacy for recurrent glioblastoma

**DOI:** 10.1038/bjc.2015.238

**Published:** 2015-06-30

**Authors:** E T Wong, E Lok, S Gautam, K D Swanson

**Affiliations:** 1 Brain Tumor Center and Neuro-Oncology Unit, Beth Israel Deaconess Medical Center, Harvard Medical School, Boston, MA 02215, USA; 2 Division of Biostatistics, Department of Medicine, Beth Israel Deaconess Medical Center, Boston, MA 02215, USA

**Keywords:** dexamethasone, glioblastoma, NovoTTF-100A, tumour immunology, chemotherapy

## Abstract

**Background:**

Patients with recurrent glioblastoma have a poor outcome. Data from the phase III registration trial comparing tumour-treating alternating electric fields (TTFields) *vs* chemotherapy provided a unique opportunity to study dexamethasone effects on patient outcome unencumbered by the confounding immune and myeloablative side effects of chemotherapy.

**Methods:**

Using an unsupervised binary partitioning algorithm, we segregated both cohorts of the trial based on the dexamethasone dose that yielded the greatest statistical difference in overall survival (OS). The results were validated in a separate cohort treated in a single institution with TTFields and their T lymphocytes were correlated with OS.

**Results:**

Patients who used dexamethasone doses >4.1 mg per day had a significant reduction in OS when compared with those who used ⩽4.1 mg per day, 4.8 *vs* 11.0 months respectively (*χ*^2^=34.6, *P*<0.0001) in the TTField-treated cohort and 6.0 *vs* 8.9 months respectively (*χ*^2^=10.0, *P*<0.0015) in the chemotherapy-treated cohort. In a single institution validation cohort treated with TTFields, the median OS of patients who used dexamethasone >4.1 mg per day was 3.2 months compared with those who used ⩽4.1 mg per day was 8.7 months (*χ*^2^=11.1, *P*=0.0009). There was a significant correlation between OS and T-lymphocyte counts.

**Conclusions:**

Dexamethasone exerted profound effects on both TTFields and chemotherapy efficacy resulting in lower patient OS. Therefore, global immunosuppression by dexamethasone likely interferes with immune functions that are necessary for the treatment of glioblastoma.

Patients with recurrent glioblastoma have limited treatment options. Bevacizumab is a standard of care for patients with recurrent glioblastoma and it produces an objective response rate of 25–60% (Wong *et al*, 2011). However, its ability to prolong patient overall survival (OS) is questionable (Iwamoto and Fine, 2010; Reardon *et al*, 2012). The NovoTTF-100A device is another FDA-approved treatment for recurrent glioblastoma that works by emitting tumour-treating alternating electric fields (TTFields) via two pairs of transducer arrays placed orthogonally on the scalp and acts to perturb tumour cells during mitosis (Kirson *et al*, 2004, 2007; Gera *et al*, 2015). Preclinical data show that cells affected by TTFields exhibit violent plasma membrane blebbing that disrupts the normal spatial ordering of the mitotic chromosomes. This results in asymmetric chromosome segregation and aneuploidy owing to defects in cytokinesis and aberrant mitotic exit. Furthermore, these cells also exhibit signs of stress that include elevated cell surface expression of calreticulin, which makes them more readily detectable by phagocytic immune cells, facilitating an immune response against the tumour (Lee *et al*, 2013). Importantly, the NovoTTF-100A device was demonstrated to possess equivalent efficacy when compared with best physician's choice (BPC) chemotherapy in the registration phase III clinical trial, but without the myeloablative toxicities associated with systemic chemotherapies that may cause secondary systemic infection or interference with immune effector function (Vecht *et al*, 1994; Hughes *et al*, 2005; Stupp *et al*, 2012; Fonkem and Wong, 2012). More recently, a prespecified interim analysis of the results from an upfront phase III clinical trial in newly diagnosed glioblastoma patients, comparing NovoTTF-100A plus adjuvant temozolomide *vs* adjuvant temozolomide alone, revealed significantly improved patient outcome with a respective progression-free survival of 7.1 *vs* 4.0 months and OS of 19.6 *vs* 16.6 months (Stupp *et al*, 2014). Compared with newly diagnosed glioblastomas, patients with recurrent glioblastoma likely have several factors that led to a worse outcome, including clonal evolution of the tumour, evasion of the immune system and reduction of immune competence because of prior exposure to chemotherapy.

Dexamethasone is commonly used to treat neurologic symptoms caused by the glioblastoma (Vecht *et al*, 1994). However, it also has a plethora of systemic toxicities, including gastrointestinal haemorrhage with or without perforation, infection, and hyperglycaemia (Heimdal *et al*, 1992). Although dexamethasone has not been shown to interfere directly with the efficacy of treatments against glioblastoma, there is emerging evidence from both preclinical and clinical data in other malignancies to suggest that dexamethasone may affect the patient's antitumour immunity. First, although the immune system has evolved multiple mechanisms to recognise and eliminate neoplastic cells (Senovilla *et al*, 2013), tumours emerge within the patient when they escape immune surveillance (Mittal *et al*, 2014). At this point, the tumour further subverts the immune system by eliciting normal wound healing and tissue remodelling responses, whereas promoting a state of immune privilege within the tumour microenvironment (Schreiber *et al*, 2011). In this setting, dexamethasone may potentiate existing local immunosuppression via global induction of I*κ*B*α* and inhibition of NF-*κ*B activity in lymphocytes, resulting in global immunosuppression (Auphan *et al*, 1995). Second, dexamethasone can lower the number of CD4^+^ lymphocytes in newly diagnosed patients with glioblastoma treated with radiation alone or in combination with temozolomide, and this attentuated CD4^+^ lymphocyte count is associated with increased infections and decreased survival (Hughes *et al*, 2005; Grossman *et al*, 2011). Lastly, recent clinical trial data have shown that there were more systemic and central nervous system responders to ipilimumab, an immune checkpoint inhibitor, in the cohort taking no dexamethasone as compared with the cohort taking dexamethasone, suggesting that dexamethasone interferes with the efficacy of ipilimumab (Margolin *et al*, 2012).

In this paper, we present evidence that immune suppression by dexamethasone markedly interferes with the clinical efficacy of two disparate therapies for recurrent glioblastoma: electric field-based therapy delivered by the NovoTTF-100A as well as conventional chemotherapies. Unlike prior clinical trials, the cohort treated with TTField monotherapy offered us an opportunity to study unambiguously the effect of dexamethasone on patient survival unencumbered by concurrent chemotherapies that suppress the immune system. We also present data that strongly support a role for immune competence in effecting TTField treatment by analysing T-cell subsets measured in a separate cohort of patients for validation.

## Patients and Methods

### Patients

Subjects signed informed consent from their respective treating institutions before participation in the phase III trial comparing NovoTTF-100A *vs* BPC chemotherapy (Fonkem and Wong, 2012; Stupp *et al*, 2012). A *post hoc* analysis of the dexamethasone effect on the two cohorts was performed based on anonymised data obtained from the sponsor, from whom the corresponding author had full access to the primary data. The outcome of the analysis was then validated retrospectively, under an institutional review board-approved protocol from Dana Farber/Harvard Cancer Center (protocol no. 12-519), using a separate cohort of patients who were treated with NovoTTF-100A and bevacizumab at Beth Israel Deaconess Medical Center.

### Statistical analysis

Statistical analyses were performed by using R statistics base package (http://www.r-project.org) and its libraries. Two-tailed Wilcoxon's rank-sum test with continuity correction was used to determine whether two independent groups of data were statistically different from each other. A modified binary search algorithm (Knuth, 1971; Tøndel *et al*, 2002), written in R, was used to iteratively partition data in both two and three dimensions. The Loess local nonparametric polynomial regression was used to perform curve fitting of the OS as a function of dexamethasone dose (Cleveland, 1979; Shipley and Hunt, 1996; Cleveland and Loader, 1996) and OS was analyzed using Kaplan–Meier statistics (Kaplan and Meier, 1958).

## Results

### Effect of dexamethasone on TTField therapy and BPC chemotherapy

**Figure 1:**
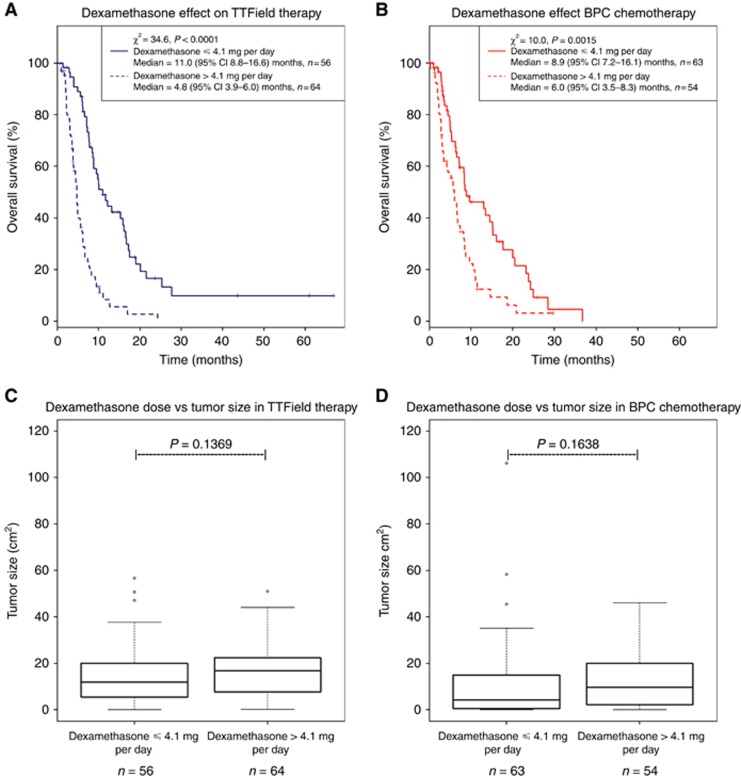
**Kaplan–Meier OS and tumour size with respect to dexamethasone requirement of ⩽4.1 *vs* >4.1 mg per day from subjects enrolled in the phase III trial comparing TTField therapy *vs* BPC chemotherapy.** (**A**) Subjects enrolled in the TTField treatment arm taking dexamethasone ⩽4.1 (solid blue) *vs* >4.1 (dashed blue) mg per day, which was determined by an unsupervised binary partitioning algorithm. Subjects who used ⩽4.1 mg per day of dexamethasone (*n*=56) had a median OS of 11.0 months (95% CI: 8.8–16.6) as compared with those who used >4.1 mg per day (*n*=64) had a median OS of 4.8 months (95% CI: 3.9–6.0) (*χ*^2^=34.6, *P*<0.0001). (**B**) Subjects enrolled in the BPC chemotherapy arm taking dexamethasone ⩽4.1 (solid red) *vs* >4.1 (dashed red) mg per day was determined by the same unsupervised binary partitioning algorithm. Subjects who used ⩽4.1 mg per day of dexamethasone (*n*=63) had a median OS of 8.9 months (95% CI: 7.2–16.1) as compared with those who used >4.1 mg per day (*n*=54) had a median OS of 6.0 months (95% CI: 3.5–8.3) (*χ*^2^=10.0, *P*=0.0015). (**C**) Box-and-whisker plot of bidimensional tumour size in the TTField therapy cohort that received dexamethasone ⩽4.1 *vs* >4.1 mg per day. Subjects who took dexamethasone ⩽4.1 mg per day (*n*=56) had a median tumour size of 11.9 (range 0.0–56.7) cm^2^ as compared with those who used >4.1 mg per day (*n*=64) had a median tumour size of 16.8 (range 0.3–51.0) cm^2^ (*P*=0.1369). (**D**) Box-and-whisker plot of bidimensional tumour size in the BPC chemotherapy cohort that received dexamethasone ⩽4.1 *vs* >4.1 mg per day. Subjects who took dexamethasone ⩽4.1 mg per day (*n*=63) had a median tumour size of 4.2 (range 0.0–11.2) cm^2^ as compared with those who used >4.1 mg per day (*n*=54) had a median tumour size of 9.6 (range 0.0–46.0) cm^2^ (*P*=0.1638).

Our previous *post hoc* analysis of responders in the phase III trial demonstrated that responders to TTField therapy required significantly lower doses of dexamethasone compared with non-responders (Wong *et al*, 2014). We therefore investigated further whether there was a threshold dose of dexamethasone that affected outcome within the entire trial population. Using an unsupervised binary partitioning algorithm (Knuth, 1971; Tøndel *et al*, 2002), we stratified the TTField therapy cohort based on the dexamethasone dose that yielded the greatest statistical difference in median OS. The results revealed that subjects who used >4.1 mg per day dexamethasone (*n*=64) exhibited a significantly shortened median OS of 4.8 months (95% confidence interval (CI): 3.9–6.0) *vs* those who used ⩽4.1 mg per day (*n*=56), with a median OS of 11.0 months (95% CI: 8.8–16.6) (*χ*^2^=34.6, *P*<0.0001; Figure 1A). We then used the same dexamethasone cutoff to stratify control patients in the BPC chemotherapy cohort and observed a similar, albeit less robust, dichotomisation, with a respective median OS of 6.0 months (95% CI: 3.5–8.3) (*n*=54) *vs* 8.9 months (95% CI: 7.2–16.1) (*n*=63) (*χ*^2^=10.0, *P*=0.0015; Figure 1B) for those receiving >4.1 *vs* ⩽4.1 mg per day of dexamethasone, respectively. There are two potential explanations for these results: either patients with larger, more aggressive tumours required a higher dose of dexamethasone for symptom control or doses of dexamethasone >4.1 mg per day interfered with both therapeutic interventions used in this trial. However, tumour size did not differ statistically between patient cohorts that used dexamethasone at either >4.1 or ⩽4.1 mg per day (Figures 1C and D). Therefore, factors other than tumour size influence the OS of subjects receiving high *vs* low doses of dexamethasone.

**Figure 2:**
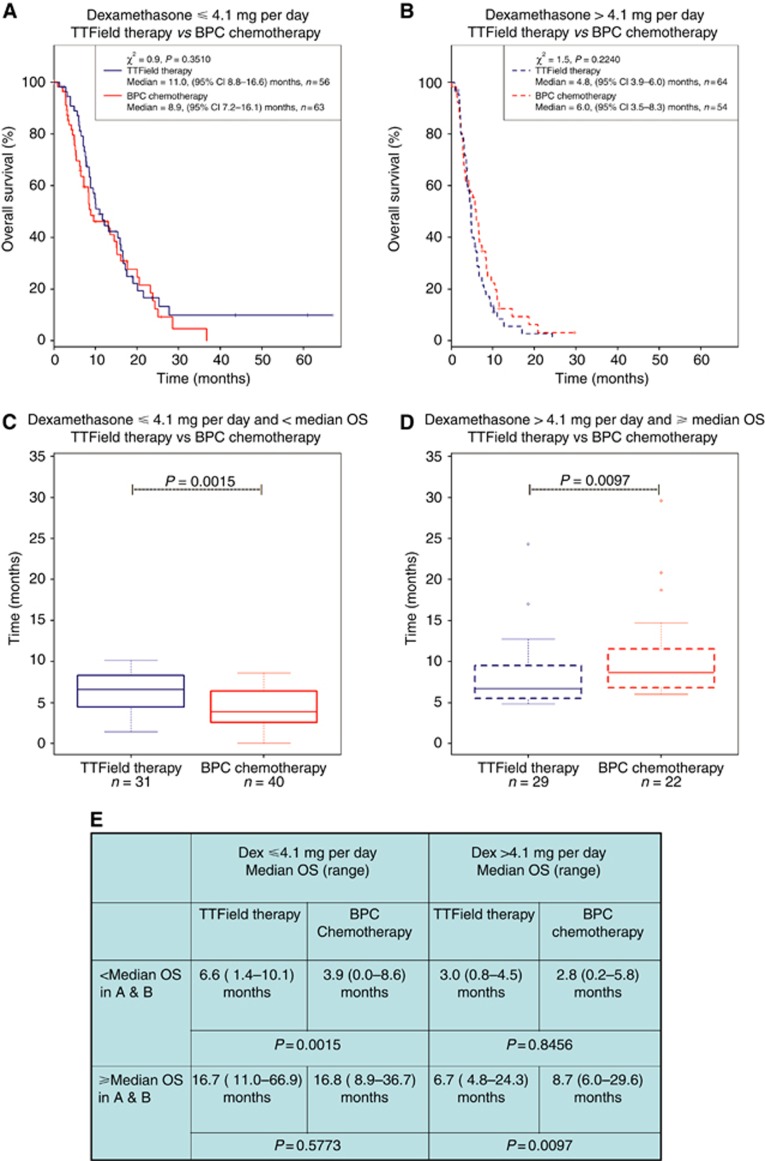
**Comparison of OS in subjects treated with TTField therapy *vs* BPC chemotherapy segregated by dexamethasone usage.** (**A**) Comparison of subjects using dexamethasone ⩽4.1 mg per day in both TTField therapy (blue) and BPC chemotherapy (red) arms. (**B**) Comparison of subjects using dexamethasone >4.1 mg per day in both TTField therapy and BPC chemotherapy arms. (**C**) Box-and-whisker plot of OS between TTField *vs* BPC chemotherapy-treated subjects using ⩽4.1 mg per day of dexamethasone and <the median OS in (**A**). The median OS was 6.6 months (range 1.4–10.1) for TTField-treated subjects (*n*=31) *vs* 3.9 months (range 0.0–8.6) for BPC chemotherapy-treated subjects (*n*=40) (*P*=0.0015). (**D**) Box-and-whisker plot of OS between TTFields *vs* BPC chemotherapy-treated subjects using >4.1 mg per day of dexamethasone and ⩾the median OS in (**B**). The median OS was 6.7 months (range 4.8–24.3) for TTField-treated subjects (*n*=29) *vs* 8.7 months (range 6.0–29.6) for BPC chemotherapy-treated subjects (*n*=22) (*P*=0.0097). (**E**) Median OS, range, and *P*-values for the four subgroups: (i) dexamethasone ⩽4.1 mg per day and <median OS in (**A)**, (ii) dexamethasone >4.1 mg per day and <median OS in (**B**), (iii) dexamethasone ⩽4.1 mg per day and ⩾median OS in (**A**), and (iv) dexamethasone >4.1 mg per day and ⩾median OS in (**B**).

To further investigate the effect of dexamethasone on patient outcome, we compared the survival characteristics of the cohort treated with TTField therapy to the one treated with BPC chemotherapy in the respective dexamethasone dosage groups. First, we compared the two treatment groups when the dosage of dexamethasone used was ⩽4.1 mg per day. Although the two OS curves overlapped (*χ*^2^=0.9, *P*=0.3510; Figure 2A), we detected a marked separation between these two curves at time points less than the median OS. Indeed, when we compared the survival curves of the two cohorts for subjects who used dexamethasone ⩽4.1 mg per day and possessed survival times of less than the median OS, we found a significant difference between the two subgroups, with a median OS of 6.6 (range 1.4–10.1) months for the TTField-treated subgroup (*n*=31) *vs* 3.9 (range 0.0–8.6) months for the BPC chemotherapy-treated subgroup (*n*=40) (*P*=0.0015; Figure 2C). However, for subjects who lived longer than the median OS, there was no difference in the OS curves, with a median OS of 16.7 (range 11.0–66.9) months for the TTField-treated subgroup (*n*=25) *vs* 16.8 (range 8.9–36.7) months for the BPC chemotherapy-treated subgroup (*n*=23) (*P*=0.5773; Figure 2E). In contrast, among subjects who received high dexamethasone doses of >4.1 mg per day, the overlapping OS curves (*χ*^2^=1.5, *P*=0.2240; Figure 2B) appeared to diverge for the subjects whose survival were greater than the median OS. Remarkably, the TTField-treated subgroup was worse compared with the BPC chemotherapy-treated subgroup when treated with dexamethasone doses >4.1 mg per day, with a respective median OS of 6.7 (range 4.8–24.3) months (*n*=29) *vs* 8.7 (range 6.0–29.6) months (*n*=22) (*P*=0.0097; Figure 2D). However, for subjects whose survival were less than the median OS and used >4.1 mg per day dexamethasone, there was no difference between the TTField-treated and the BPC chemotherapy-treated subgroups, with the former having a median OS of 3.0 (range 0.8–4.5) months (*n*=35) as compared with the latter having a median OS of 2.8 (range 0.2–5.8) months (*n*=32) (*P*=0.8456; Figure 2E). Collectively, the data in Figures 2C and D indicate that the extent of dexamethasone exposure not only predicted treatment efficacy but also strongly suggest that TTField therapy is superior to BPC chemotherapy in the setting of low dexamethasone usage. However, under the influence of higher dexamethasone usage, the benefit of TTField therapy appeared to be negated to a greater extent when compared with BPC chemotherapy as if TTField-treated subjects were not provided with any therapy at all.

### Dose-dependent effect of dexamethasone on treatment efficacy

**Figure 3:**
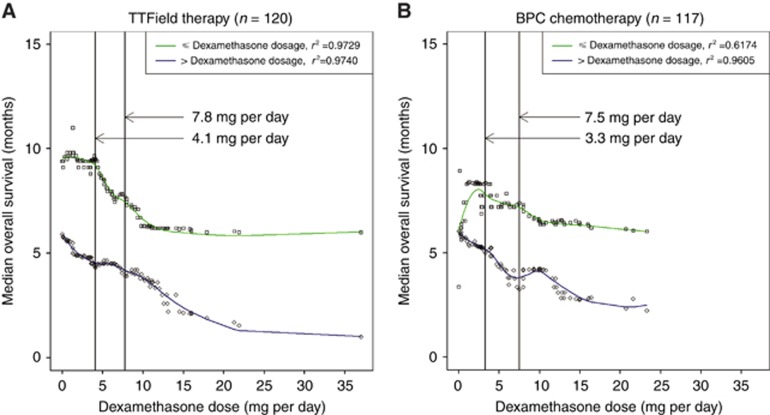
**Loess local polynomial regression of median OS *vs* dexamethasone dose.** Dexamethasone was treated as a discrete variable successively and the median OS was plotted for the group ⩽(green) and >(blue) compared with the variable dosage of dexamethasone. Curve fitting was performed using the Loess local polynomial regression. (**A**) In the TTField therapy cohort (*n*=120), there was decremental OS from 4.1 mg per day that reached an inflection point at 7.8 mg per day, after which the rate of OS decrease slowed. (**B**) In the BPC chemotherapy cohort (*n*=117), there was decremental OS from 3.3 mg per day that reached an inflection point at 7.5 mg per day, after which the rate of OS decrease slowed.

We next asked whether or not dexamethasone has a dose-dependent influence on treatment efficacy by analysing the entire dose spectrum used in the trial. We partitioned the TTField-treated cohort using a dexamethasone dose cutoff from 0.0 to 37.0 mg per day, plotted the respective median OS of the groups at ⩽cutoff or >cutoff *vs* successive dexamethasone dosages, and fitted the data with the best curves using the nonparametric Loess local polynomial regression (Figure 3) (Cleveland, 1979; Cleveland and Loader, 1996; Shipley and Hunt, 1996). In addition, we plotted the log-rank *P*-values of the dichotomised groups in each successive dexamethasone dosage and found two nadir *P*-values of 0.00000008 and 0.00002524 corresponding to dexamethasone doses of 4.1 and 7.8 mg per day, respectively. We observed that there was decremental OS starting at a dexamethasone dose of 4.1 mg per day and, with successive increases of dexamethasone, reached an inflection point at 7.8 mg per day, after which the rate of OS decreased slowly (Figure 3A).

We also performed the same dose-dependent analysis of dexamethasone in the BPC chemotherapy-treated cohort and found a nadir *P*-value of 0.00163291 at 3.3 mg per day and another of 0.00011858 at 7.5 mg per day. Similarly, the best-fit curve derived in Figure 3B also suggests that the dexamethasone dose near 4 mg per day may also represent a point at which decremental OS can be observed with successive increases in dexamethasone dosage. This progressive decrement in OS occurred with successive increases of dexamethasone until an inflection point is observed at a dose near 7.5 mg per day, after which the rate of OS decreased slowly. Taken together, both cohorts experienced interference from dexamethasone at a dose near 4.0 mg per day and a maximal effect was observed near 7.5 mg per day.

### Validation of the dexamethasone effect on TTField-treated patients from a single institution

**Figure 4:**
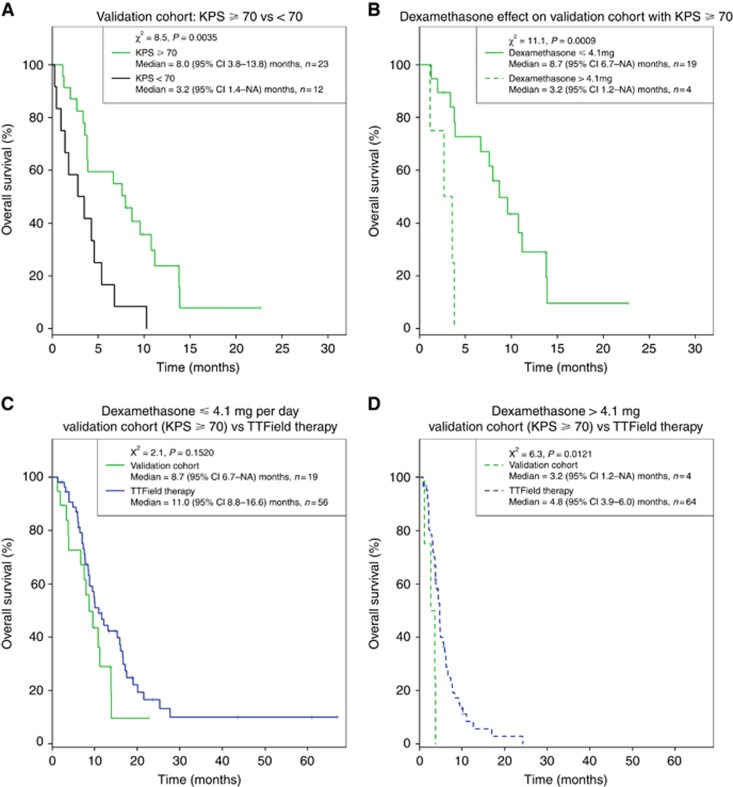
**Kaplan–Meier estimates of survival in the validation cohort from a single institution.** (**A**) The Kaplan–Meier survival curves for patients with KPS ⩾70 (solid green) *vs* those with KPS <70 (solid black). (**B**) Dexamethasone effect on the cohort with KPS ⩾70 by comparing patients taking dexamethasone ⩽4.1 (solid green) *vs* those taking >4.1 mg per day (dashed green). (**C**) Comparison of the TTField-treated subjects who used ⩽4.1 mg per day of dexamethasone in the phase III trial (from Figure 2A) *vs* the validation cohort with having KPS ⩾70 and taking dexamethasone ⩽4.1 mg per day. (**D**) Comparison of the TTField-treated subjects who used >4.1 mg per day of dexamethasone in the phase III trial (from Figure 2B) *vs* the validation cohort with having KPS ⩾70 and taking dexamethasone >4.1 mg per day.

We next proceeded to validate the observed dexamethasone effect on patient outcome within the trial by retrospectively analysing our own single-institution cohort. From November 2012 to February 2014, we treated 38 patients (Table 1) using TTField monotherapy as treatment or in combination with bevacizumab, whereas dexamethasone usage was aggressively reduced. Three patients who were referred specifically to our institution did not receive TTField therapy because of patient choice of other treatments, severe medical comorbidities, or advanced intracranial disease that was deemed more suitable for hospice care. Among the remaining 35 patients, their median OS was 4.3 months (95% CI: 3.5–8.7). To properly compare this cohort with the subjects enrolled in the phase III trial, we included only those with a KPS ⩾70 or greater (*n*=23) in our validation set. This sub-population exhibited a median OS of 8.0 months (95% CI: 3.8–13.8) compared with 3.2 months (95% CI: 1.4–NA) for the remaining patients with a KPS <70 (*n*=12) (*χ*^2^=8.5, *P*=0.0035; Figure 4A). We then applied a cutoff of dexamethasone 4.1 mg per day as was found in our previous binary partitioning analysis. Patients who used dexamethasone ⩽4.1 mg per day had a significantly longer OS compared with those who used >4.1 mg per day, with a median OS of 8.7 months (95% CI: 6.7–NA) (*n*=19) *vs* 3.2 months (95% CI: 1.2–NA) (*n*=4), respectively (*χ*^2^=11.1, *P*=0.0009; Figure 4B). Although our single-institution cohort has fewer patients compared with the cohorts in the phase III trial, we nevertheless observed a robust segregation of OS in the patient groups, validating the previously observed effect of dexamethasone on patient outcome.

Comparison of patients within the validation cohort with a KPS ⩾70 and dexamethasone usage ⩽4.1 mg per day (*n*=19) to the phase III TTField therapy cohort who used dexamethasone ⩽4.1 mg per day (*n*=56, from Figure 2A) revealed no statistical difference between the two groups, with a median OS of 8.7 months (95% CI: 6.7–NA) *vs* 11.0 months (95% CI: 8.8–16.6), respectively (*χ*^2^=2.1, *P*=0.1520; Figure 4C). We next asked whether important prognostic factors within our cohort varied relative to patients within the phase III cohort by examining the possible effects of age and tumour size. The median age of our cohort was 57 (range 30–77) years and it is not different from the median age of 54 (range 24–80) years in the TTField-treated cohort from the phase III trial (Stupp *et al*, 2012). Average tumour size in our cohort as measured by gadolinium-enhanced T1-weighted MRI showed a median bidimensional measurement of 12.2 (range 0.30–40.6) cm^2^, which is similar to the median bidimensional measurement of 14.2 (0.0–56.7) cm^2^ in the TTField-treated phase III cohort (*P*=0.6178; Table 1). However, 15 of 23 patients (65%) were already on bevacizumab before their neuroimaging studies, possibly interfering with tumour measurement because bevacizumab can reduce vascular permeability in tumours causing decreased gadolinium enhancement (Wong and Brem, 2008). Further, blockade of vascular endothelial growth factor can promote an invasive and diffuse glioblastoma phenotype that result in tumours possessing greater size than can be measured on gadolinium-enhanced T1-weighted MRI (Norden *et al*, 2008; Lu *et al*, 2012). We therefore measured the bidimensional size of the FLAIR abnormality. Indeed, in our cohort, the median bidimensional FLAIR abnormality was 29.6 (range 7.0–60.2) cm^2^, which is more than two times the tumour size observed on gadolinium-enhanced T1-weighted MRI in the phase III trial (Stupp *et al*, 2012). As expected, this bevacizumab effect on tumour measurement was corroborated in our entire patient cohort (*n*=38) by the strong correlation between the size of the gadolinium-enhanced T1-weighted and FLAIR measured bidimensional tumour size among those not on bevacizumab (*r*^2^=0.7333, *n*=10; Supplementary Figure 1A), whereas no such correlation was seen among those on bevacizumab (*r*^2^=0.1446, *n*=27; Supplementary Figure 1B). Furthermore, we found that patients in our validation cohort who used dexamethasone >4.1 mg per day (*n*=4) had a worse outcome compared with the corresponding cohort in the phase III trial (*n*=64), with a median OS of 3.2 months (95% CI: 1.2–NA) *vs* 4.8 months (95% CI: 3.9–6.0), respectively (*χ*^2^=6.3, *P*=0.0121; Figure 4D). Therefore, our single-institution validation cohort, who had KPS ⩾70, used dexamethasone ⩽4.1 mg per day and possessed greater tumour burden, compared favourably with those treated with TTFields therapy in the phase III trial, but those with KPS ⩾70 but used dexamethasone >4.1 mg per day probably suffered from a worse outcome compared with the corresponding trial cohort.

### Patient immune characteristics and TTField therapy efficacy

**Figure 5:**
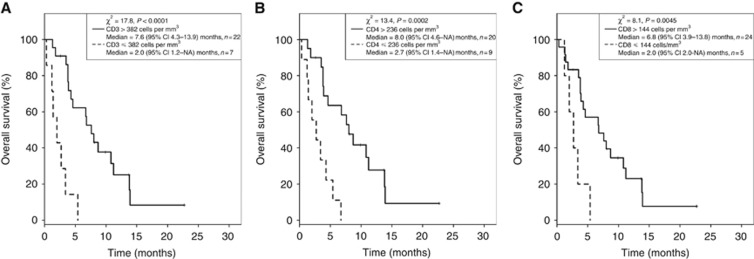
**Wilcoxon's rank-sum test of the optimal cutoff T-lymphocyte subsets as determined by an unsupervised binary partitioning algorithm.** (**A**) Median OS of patients with absolute CD3^+^ ⩽382 *vs* >382 cells per mm^3^ was 2.0 months (range 0.3–5.4) (*n*=7) and 7.7 months (range 1.3–22.7) (*n*=25), respectively (*P*=0.0017). (**B**) Median OS of patients with absolute CD4^+^ ⩽236 *vs* >236 cells per mm^3^ was 2.7 months (range 0.3–6.7) (*n*=9) and 8.0 months (range 1.3–22.7) (*n*=23), respectively (*P*=0.0029). (**C**) Median OS of patients with absolute CD8^+^ ⩽144 *vs* >144 cells per mm^3^ was 2.7 months (range 1.2–5.4) (*n*=5) and 7.6 months (range 0.3–22.7) (*n*=27), respectively (*P*=0.0313).

Dexamethasone has been associated with profound immunosuppression (Hughes *et al*, 2005; Grossman *et al*, 2011) and it may severely limit a patient's ability to mount an antitumour immune response against the glioblastoma (Zitvogel *et al*, 2008a). Our data clearly demonstrated that dexamethasone doses higher than a threshold level of 4.1 mg per day correlated with a poorer patient outcome during TTField therapy. This finding strongly suggests an immunological component behind the efficacy of this intervention and that factors required for general immune competence may have a role in predicting therapeutic outcome in our patients. We therefore analysed their CD3^+^, CD4^+^, and CD8^+^ T-lymphocyte subsets during the course of their treatment. Using the unsupervised binary partitioning approach described above for dexamethasone dose, we attempted to identify whether there was any threshold for the absolute CD3^+^, CD4^+^, or CD8^+^ T-lymphocyte count, which yielded the greatest statistical difference in OS when used to stratify our patient population. Significantly, this analysis revealed that the median OS of patients with absolute CD3^+^ ⩽382 cells per mm^3^ was 2.0 months (95% CI: 1.2–NA) (*n*=7). In contrast, the median OS of those with CD3^+^ >382 cells per mm^3^ was 7.6 months (95% CI: 4.3–13.9) (*n*=22) (*χ*^2^=17.8, *P*<0.0001; Figure 5A), with the data showing that patient survival was positively correlated with the absolute numbers of CD3^+^ T lymphocytes. Similarly, we found that patients with absolute CD4^+^ ⩽236 cells per mm^3^ exhibited a median OS of 2.7 months (95% CI: 1.4–NA) (*n*=9) as compared with those with CD4^+^ >236 cells per mm^3^ with a median OS of 8.0 months (95% CI: 4.6–NA) (*n*=20) (*χ*^2^=13.4, *P*=0.0002; Figure 5B). Furthermore, patients with an absolute CD8^+^ count of ⩽144 cells per mm^3^ exhibited a median OS of 2.0 months (95% CI: 2.0–NA) (*n*=5) as compared with 6.8 months (95% CI: 3.9–13.8) (*n*=24) for those with CD8^+^ >144 cells per mm^3^ (*χ*^2^=8.1, *P*=0.0045; Figure 5C).

We next asked whether CD3^+^, CD4^+^, and CD8^+^ lymphocyte counts was related to the overall status of the patient's peripheral blood counts and dexamethasone requirement. As expected, there was a correlation between C3^+^ and CD4^+^ cells (*r*^2^=0.6949) and between CD3^+^ and CD8^+^ cells (*r*^2^=0.5001) but not between CD4^+^ and CD8^+^ cells (*r*^2^=0.0733). However, there was no correlation between white blood cells (WBC) and CD3^+^ cells (*r*^2^=0.0053), WBC and CD4^+^ cells (*r*^2^=0.0023), and WBC and CD8^+^ cells (*r*^2^=0.0032). No correlation was also detected between platelets and CD3^+^ cells (*r*^2^=0.2576), platelets and CD4^+^ (*r*^2^=0.2746), and platelets and CD8^+^ (*r*^2^=0.0887). Similarly, there was no correlation between the daily dexamethasone dose and CD3^+^ cells (*r*^2^=0.1888), dexamethasone and CD4^+^ cells (*r*^2^=0.1531), and dexamethasone and CD8^+^ cells (*r*^2^=0.0451). Taken together, CD3^+^, CD4^+^, and CD8^+^ lymphocyte counts appear to be independent of the peripheral blood counts and dexamethasone dose effect. Therefore, T-lymphocyte counts may serve as an independent measure of immunocompetence in our patients and predict treatment outcome when using NovoTTF-100A.

## Discussion

Our previous *post hoc* analysis of responders in the phase III trial comparing NovoTTF-100A monotherapy and BPC chemotherapy for recurrent glioblastoma revealed that dexamethasone and prior low-grade glioma histology were predictors of response (Wong *et al*, 2014). Traditionally, oncologists view dexamethasone's influence on glioblastoma patients from the perspective of its antioedema effect from the tumour (Vecht *et al*, 1994), antiemetic efficacy against emetogenic chemotherapies, infections from its systemic immunosuppressive property (Vecht *et al*, 1994; Hughes *et al*, 2005), and changes in contrast enhancement on computed tomography (Chamberlain *et al*, 1988) or MRI (Ostergaard *et al*, 1999). Because dexamethasone has the potential to produce profound toxicities in patients in large part by suppressing their immune system and it is a clinically modifiable factor, we therefore extended our analysis of possible dexamethasone effect on outcome to the entire trial cohort. In this study, we have uncovered compelling evidence that dexamethasone counteracted the therapeutic efficacy of TTFields. Further, we also found that its use negatively correlated with survival in the cohort treated with chemotherapy. Our analysis is the first to show this significant impact of dexamethasone on treatment efficacy and patient OS, which is a discrete and unequivocal endpoint in contrast to progression-free survival or response for the conduct of clinical trials for recurrent glioblastomas.

In contrast to commonly used chemotherapeutic regimens, TTField monotherapy does not exert deleterious effects on the immune system, and thus, unlike the chemotherapy-treated cohort, TTField-treated subjects did not receive concurrent immunosuppressive agents other than dexamethasone during the entire trial period. Therefore, this trial provided us with a unique opportunity to examine the interference of dexamethasone on the clinical outcome of patients without the confounding influence of cytotoxic chemotherapies. Given our previous observation that responders from this trial had low dexamethasone usage (Wong *et al*, 2014), we first asked whether we could determine a threshold of dexamethasone exposure below which a benefit in patient survival could be detected within the entire cohort. Using an unsupervised mathematical algorithm, we found that a dexamethasone dose of 4.1 mg per day produced the greatest statistical segregation of OS in the TTField-treated cohort, and subjects who received >4.1 mg per day had a 2.3-fold decrease in median OS compared with those who used ⩽4.1 mg per day. Notably, using this dose level to stratify the control cohort treated with BPC chemotherapy also produced a statistically significant, but less robust, OS segregation, and subjects who received >4.1 mg per day had a 1.5-fold decrease in median OS compared with those who used ⩽4.1 mg per day. Within both cohorts, patients exhibited a decrease in OS starting at about 4.0 mg per day, with progressive decrement until a dosage of 8.0 mg per day, above which there was no further decrease in OS. Therefore, our data indicate that dexamethasone has a generalised and profound interference on treatment efficacy regardless of whether the treatment has non-cytotoxic or cytotoxic properties on the haematopoietic system.

Our analysis strongly indicates that dexamethasone interferes with the efficacy of both TTFields and BPC chemotherapies, the latter of which consisted largely of alkylating chemotherapies. In the sub-populations taking ⩽4.1 mg per day of dexamethasone, 31 subjects treated with TTField monotherapy exhibited a better outcome compared with the corresponding 40 subjects treated with BPC chemotherapy. This small but statistically significant benefit occurred within the first 11 months, after which the OS of the two cohorts overlapped and the benefit from TTField therapy dissipated. In contrast, for the sub-population taking >4.1 mg per day of dexamethasone, 29 subjects treated with TTField monotherapy exhibited a worse outcome relative to the corresponding 22 subjects treated with BPC chemotherapy. Therefore, high dexamethasone dosage appears to negate or counteract the effect of both TTField therapy and BPC chemotherapy. Because the overall trial population in the TTField-treated cohort is only 120, the benefit of treatment in the 31 (26%) subjects taking ⩽4.1 mg per day of dexamethasone is essentially negated by the hindrance caused by the 29 (24%) patients taking >4.1 mg per day of dexamethasone when the populations were not segregated based on dexamethasone burden. This dexamethasone interference with TTField efficacy may explained the improved outcome seen in the trial for newly diagnosed glioblastoma patients (Stupp *et al*, 2014), who were not as severely affected by treatment effects when compared with recurrent glioblastoma patients who had a longer exposure to cytotoxic chemotherapy, dexamethasone, or both.

Our data also indicate that T-lymphocyte subsets may have an important role in the outcome of our validation cohort of patients treated with TTField therapy, with prolonged OS associated with absolute CD3^+^ >382 cells per mm^3^, CD4^+^ >235 cells per mm^3^, and CD8^+^ >144 cells per mm^3^ in an unsupervised analysis. Hughes *et al* (2005) and Grossman *et al* (2011) both showed that dexamethasone induces a drop in CD4^+^ lymphocyte count, which predisposes glioblastoma patients to infectious complications, and a CD4^+^ count <200 cells per mm^3^ is associated with poor survival. However, we also noted that dexamethasone's immunosuppressive effect also blunted the therapeutic efficacy of TTField therapy and chemotherapy, probably as a result of its global interference with the patient's immune system. This notion is supported by our *in vitro* experiments, which demonstrated that cells attempting to divide in the presence of the TTFields are disrupted in mitosis during the metaphase-to-anaphase transition and experienced aberrant mitotic exit (Gera *et al*, 2015). These cells subsequently exhibited changes consistent with immunogenic cell death and thus were susceptible to immune elimination (Lee *et al*, 2011, 2013). Because subjects that received dexamethasone ⩽4.1 mg per day in the phase III trial exhibited benefit from TTField therapy, the observed benefit is strongly consistent with an increased immunogenicity of cells affected by TTFields. Furthermore, a number of cytotoxic chemotherapy agents, such as doxorubicin, 5-fluorouracil, and oxaliplatin, can induce either genomic or cytoplasmic stress in the tumour cell leading to immunogenic cell death (Zitvogel *et al*, 2008b). Although the extent of immunostimulatory effects of alkylators, such as lomustine, carmustine, procarbazine, and temozolomide is unknown, dacarbazine has been shown to upregulate NKG2D ligands on tumour cells and thereby target them for immune elimination by natural killer (NK) cells and CD8^+^ cytotoxic T-lymphocytes (Hervieu *et al*, 2013). Furthermore, alkylating agents have been shown to induce the secretion of ATP and HMGB1, both of which are danger signals that can activate immune responses against dying cells (Zong *et al*, 2004). Lastly, in myeloma patients, dexamethasone can severely block lenolidomide-induced NK cell activation (Hsu *et al*, 2011). Taken together, there is a strong indication from our data that the cytotoxic agents used in the trial against recurrent glioblastomas also act by inducing immune responses against the tumour and that concurrent dexamethasone usage negated this benefit.

There are a number of limitations in the interpretation of our findings. First, our data only allowed us to examine global immunosuppression in our patients but provide no means to assess local immunosuppression within the tumour microenvironment. This local suppression of immune surveillance is thought to be mediated by arginase, regulatory T cells, and myeloid-derived immunosuppressive cells (Fecci *et al*, 2006; Jacobs *et al*, 2010; Raychaudhuri *et al*, 2011). Nevertheless, removal of global immunosuppressive factors is the first step towards successful antiglioblastoma therapy. Second, our T-lymphocyte analysis only measured cells in the adaptive immune system. However, TTField therapy and certain chemotherapy agents could potentially induce an NK cell response against the glioblastoma (Hervieu *et al*, 2013; Lee *et al*, 2013). However, the observed dexamethasone effect on absolute CD3^+^, CD4^+^, and CD8^+^ lymphocytes could also negatively influence the activation of other cytotoxic subsets such as NK cells (Hsu *et al*, 2011). Therefore, future analysis of the specific effects of dexamethasone on glioblastoma treatment would need to include the global effect on these cells.

In conclusion, dexamethasone exerted a profound interference on the therapeutic effects of both TTField therapy and BPC chemotherapies. The threshold dose at which dexamethasone was able to be used with minimal interference on these treatments was 4.1 mg per day or lower. In our validation set of TTField-treated patients, the cluster that had the longest OS had CD3^+^ >382 cells per mm^3^, CD4^+^ >236 cells per mm^3^, and CD8^+^ >144 cells per mm^3^. Taken together, these data strongly suggest that the stimulation of immunity against the tumour operates in both of these therapeutic approaches. Future clinical trials for recurrent glioblastoma, as well as other types of brain tumours, may need to take into account the influence of dexamethasone on therapeutic outcome.

## Figures and Tables

**Table 1:** Patient characteristics in the validation cohort and the NovoTTF-100A cohort in phase III trial

**Patient characteristics**	**Validation cohort (*****n*****=35)**	**NovoTTF-100A cohort (*****n*****=120)**	***P*****-value**
Age (range)	57 (30−77) years	54 (24–80) years	
**Gender**			
Male	22 (63%)	92 (77%)	
Female	13 (37%)	28 (23%)	
**Karnofsky performance status**			
Median	70 (range 50–90)	80 (range 50–100)	
**Tumour size, bidimensional**			
T1 Gad, median (range) (cm^2^)	12.2 (0.3−40.6)	14.2 (0.0–56.7)	0.6178
FLAIR, median (range) (cm^2^)	35.2 (7.0−90.9)	N/A	
**Dexamethasone dose**			
Median (range) (mg per day)	3.0 (0.0−15.0)	4.7 (0.0–37.5)	
**Absolute T-cell subsets**			
CD3, median (range) (cells per mm^3^)	733 (70−1458)	N/A	
CD4, median (range) (cells per mm^3^)	414 (25−788)	N/A	
CD8, median (range) (cells per mm^3^)	302 (44−1039)	N/A	
**Prior therapy**			
First recurrence	6 (17%)	11 (9%)	
Second recurrence	10 (29%)	58 (48%)	
Third recurrence	19 (54%)	51 (43%)	
Prior bevacizumab	25 (71%)	23 (19%)	
**Outcome**			
Overall survival, median (months)	4.3 (95% CI: 3.5–8.7)	7.1 (95% CI: 6.1–8.8)	0.0468

Abbreviations: CI=confidence interval; FLAIR=fluid-attenuated inversion recovery; Gad=gadolinium; N/A=not applicable; TTF=tumour-treating alternating electric field.
