# Supplementary figures and images for: Dexamethasone exerts profound immunologic interference on treatment efficacy for recurrent glioblastoma
Source: Br J Cancer. 2015 Jun 30;113(2):232–41. doi: 10.1038/bjc.2015.238 (PMC4506397; doi:10.1038/bjc.2015.238)

Supplemental Figure 1

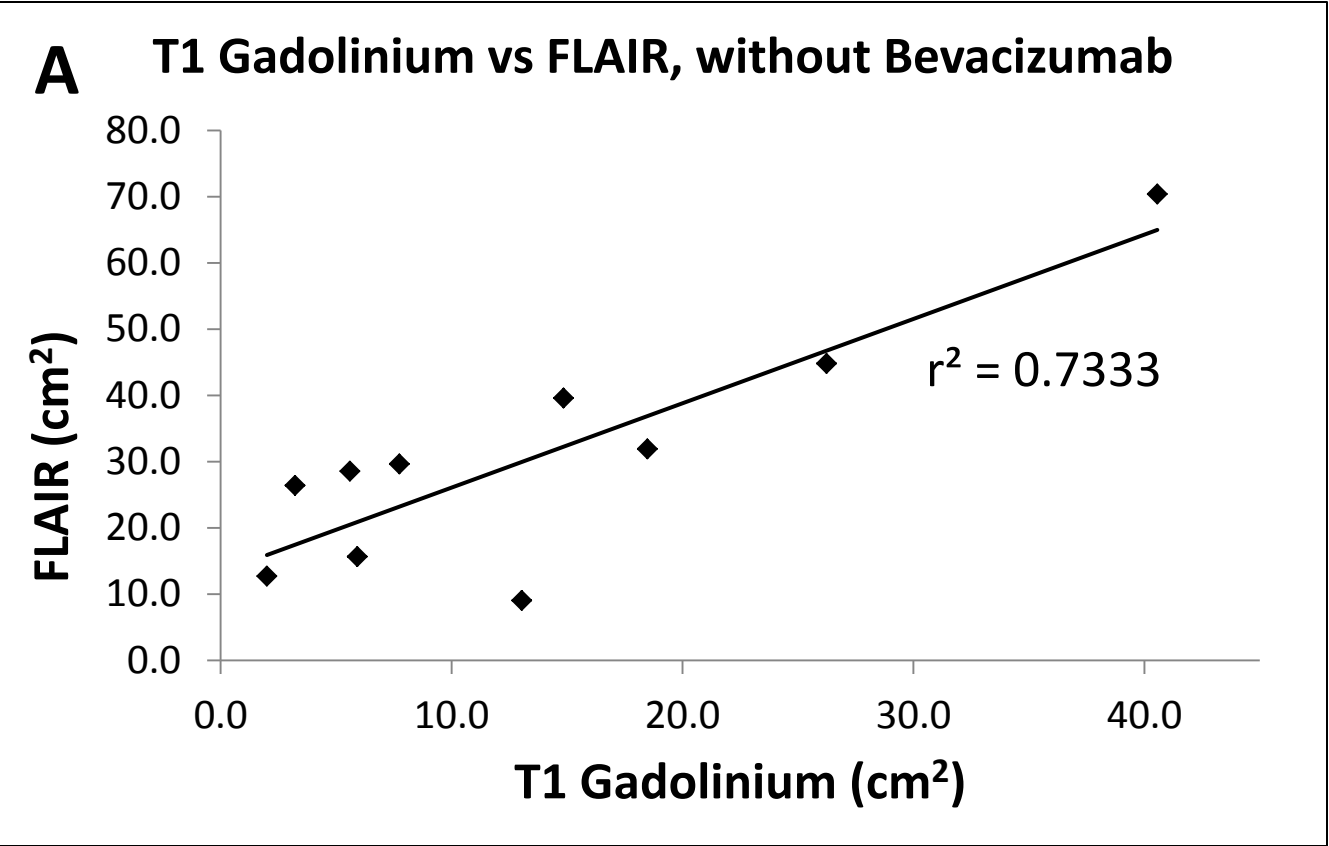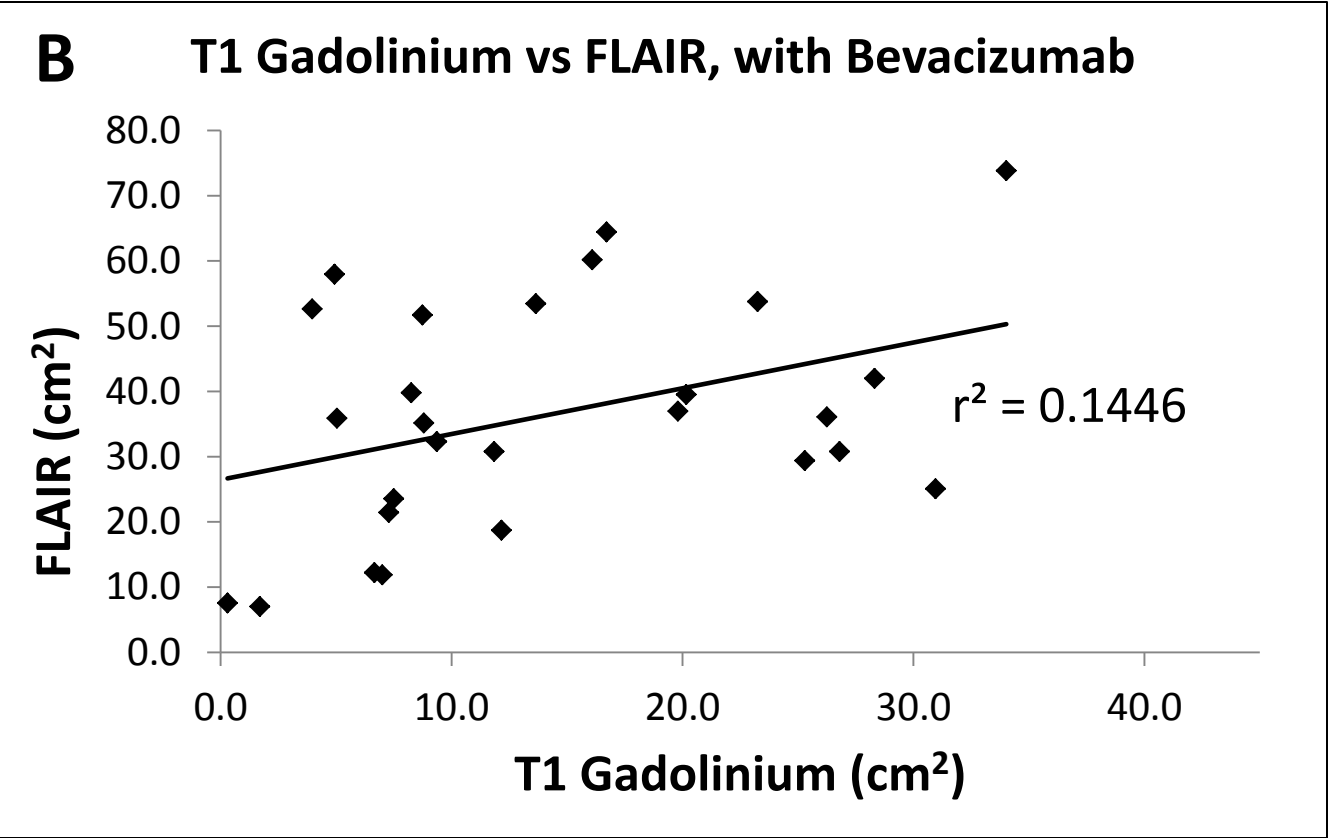

Supplement: Supplementary Information [file bjc2015238x1.pdf]
